# 4-Cyano-1-methyl­pyridinium perchlor­ate

**DOI:** 10.1107/S1600536814012860

**Published:** 2014-06-07

**Authors:** Vu D. Nguyen, Cameron A. McCormick, Lynn V. Koplitz, Joel T. Mague

**Affiliations:** a Department of Chemistry, Loyola University, New Orleans, LA 70118, USA; b Department of Physics, Loyola University, New Orleans, LA 70118, USA; c Department of Chemistry, Tulane University, New Orleans, LA 70118, USA

## Abstract

The title salt, C_7_H_7_N_2_^+^·ClO_4_^−^, crystallizes with alternating cations and anions in wavy sheets, which are formed by a number of C—H⋯O and C—H⋯N hydrogen bonds, lying approximately parallel to (001).

## Related literature   

For the crystal structures of other 4-cyano-1-methyl­pyridinium salts, see: McCormick *et al.* (2013 ▶); Kammer *et al.* (2012*a* ▶,*b* ▶); Hardacre *et al.* (2008 ▶, 2010 ▶); Glavcheva *et al.* (2004 ▶); Bockman & Kochi (1989 ▶, 1992 ▶). For the structure of 3-cyano-1-methyl­pyridinium perchlorate, see: McCormick *et al.* (2014 ▶) and for the structure of 4-cyano­anilinium perchlorate, see: Dai (2008 ▶). For a discussion of anion–π interactions, see: Frontera *et al.* (2011 ▶).
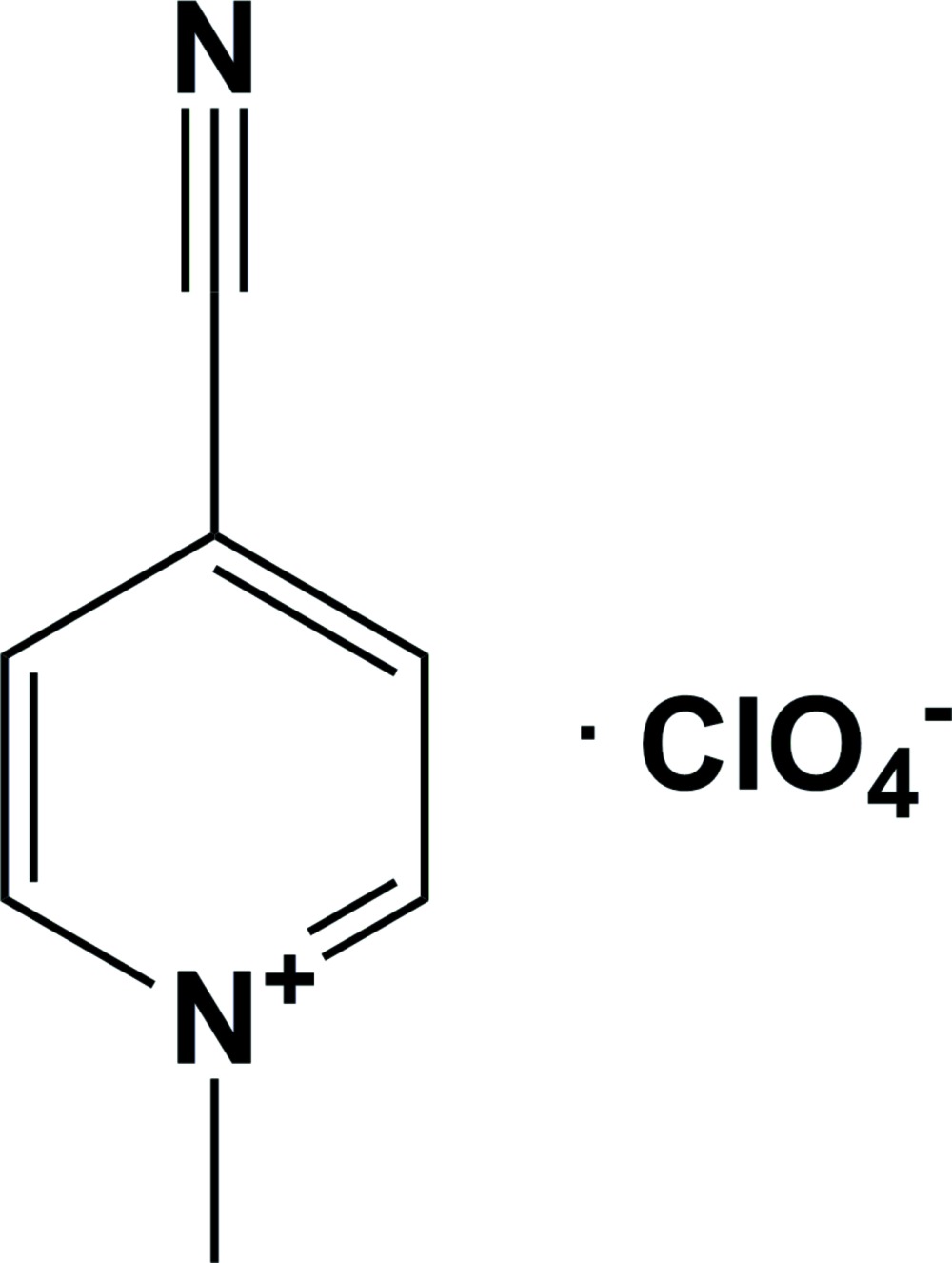


## Experimental   

### 

#### Crystal data  

C_7_H_7_N_2_^+^·ClO_4_^−^
*M* *_r_* = 218.60
Orthorhombic, 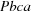

*a* = 10.232 (2) Å
*b* = 10.872 (3) Å
*c* = 16.769 (4) Å
*V* = 1865.3 (7) Å^3^
*Z* = 8
Mo *K*α radiation
μ = 0.40 mm^−1^
*T* = 100 K
0.23 × 0.16 × 0.12 mm

#### Data collection  

Bruker SMART APEX CCD diffractometer
Absorption correction: multi-scan (*SADABS*; Bruker, 2010 ▶) *T*_min_ = 0.86, *T*_max_ = 0.95
30647 measured reflections
2475 independent reflections
2235 reflections with *I* > 2σ(*I*)
*R*_int_ = 0.054

#### Refinement  

*R*[*F* ^2^ > 2σ(*F* ^2^)] = 0.034
*wR*(*F* ^2^) = 0.097
*S* = 1.07
2475 reflections
129 parameters
H-atom parameters constrained
Δρ_max_ = 0.38 e Å^−3^
Δρ_min_ = −0.39 e Å^−3^

### 

Data collection: *APEX2* (Bruker, 2010 ▶); cell refinement: *SAINT* (Bruker, 2010 ▶); data reduction: *SAINT*; program(s) used to solve structure: *SHELXS97* (Sheldrick, 2008 ▶); program(s) used to refine structure: *SHELXL97* (Sheldrick, 2008 ▶); molecular graphics: *DIAMOND* (Brandenburg & Putz, 2012 ▶); software used to prepare material for publication: *SHELXTL* (Sheldrick, 2008 ▶)’).

## Supplementary Material

Crystal structure: contains datablock(s) I, global. DOI: 10.1107/S1600536814012860/su2740sup1.cif

Structure factors: contains datablock(s) I. DOI: 10.1107/S1600536814012860/su2740Isup2.hkl

Supporting information file. DOI: 10.1107/S1600536814012860/su2740Isup3.cml

CCDC reference: 1006394

Additional supporting information:  crystallographic information; 3D view; checkCIF report

## Figures and Tables

**Table 1:** Hydrogen-bond geometry (Å, °)

*D*—H⋯*A*	*D*—H	H⋯*A*	*D*⋯*A*	*D*—H⋯*A*
C1—H1*A*⋯O4^i^	0.98	2.37	3.245 (2)	149
C1—H1*C*⋯O2^ii^	0.98	2.61	3.2540 (19)	123
C2—H2⋯O1^ii^	0.95	2.46	3.4001 (18)	173
C2—H2⋯O2^ii^	0.95	2.63	3.2549 (18)	123
C3—H3⋯N2^iii^	0.95	2.67	3.3098 (18)	125
C3—H3⋯O3^iv^	0.95	2.46	3.300 (2)	148
C6—H6⋯O4^i^	0.95	2.50	3.351 (2)	149

Symmetry codes: (i) 
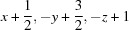
; (ii) 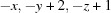; (iii) 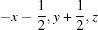; (iv) 
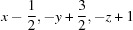
.

**Fig. 1.**
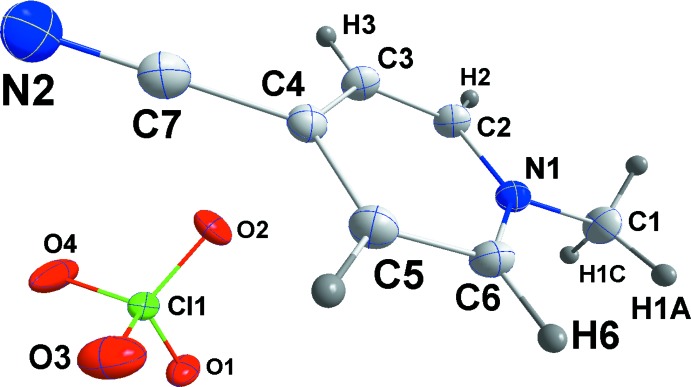
A view of the molecular structure of the title compound, with atom labelling. Displacement ellipsoids are drawn at the 50% probability level.

**Fig. 2.**
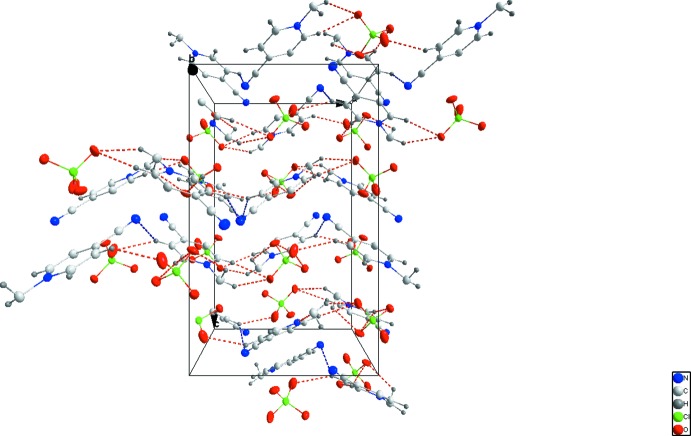
A view of the crystal packing along the b axis, with the C—H···O and C—H···N hydrogen bonds as red and blue dashed lines, respectively (see Table 1 for details).

## supplementary crystallographic information

### S1. Comment

The title compound, Fig. 1, crystallizes with alternating cations and anions in wavy sheets, which are formed by a number of C—H···O and C—H···N hydrogen bonds, which are approximately parallel to (001) [see Table 1 and Fig. 2].

As with 3-cyano-1-methylpyridinium perchlorate (McCormick *et al.*, 2014), the perchlorate ions are located near the pyridinium nitrogen atoms as the result of electrostatic attraction but the remainder of the two structures differ considerably due to the different position of the cyano group and the effect this has on the weak interionic interactions.

### S2. Experimental

4-Cyanopyridine (10.55 g) was dissolved in benzene (40 ml). Iodomethane (9.5 ml) was added to this solution slowly with stirring and the solution was refluxed for 75 minutes. Yellow solid 4-cyano-1-methylpyridinium iodide (m.p. 189–193° C) was collected by vacuum filtration. This solid (0.98 g) was then dissolved in a solution of silver perchlorate previously prepared by reacting Ag_2_O (0.47 g) with 0.5 *M* aqueous HClO_4_(8.0 ml). After stirring, precipitated AgI was removed by vacuum filtration and the filtrate containing 4-cyano-1-methylpyridinium perchlorate (m.p.114–119° C) was slowly evaporated to dryness to form crystals of the title compound.

### S3. Refinement

H-atoms were placed in calculated positions (C—H = 0.95 - 0.98 Å) and included as riding contributions with U_iso_(H) = 1.5U_eq_(C-methyl) and = 1.2U_eq_(C) for other H atoms.

### Crystal data

**Table:**

C_7_H_7_N_2_^+^·ClO_4_^−^	*F*(000) = 896
*M**_r_* = 218.60	*D*_x_ = 1.557 Mg m^−^^3^
Orthorhombic, *P**b**c**a*	Mo *K*α radiation, λ = 0.71073 Å
Hall symbol: -P 2ac 2ab	Cell parameters from 9543 reflections
*a* = 10.232 (2) Å	θ = 3.0–29.1°
*b* = 10.872 (3) Å	µ = 0.40 mm^−^^1^
*c* = 16.769 (4) Å	*T* = 100 K
*V* = 1865.3 (7) Å^3^	Block, colourless
*Z* = 8	0.23 × 0.16 × 0.12 mm

### Data collection

**Table:**

Bruker SMART APEX CCD diffractometer	2475 independent reflections
Radiation source: fine-focus sealed tube	2235 reflections with *I* > 2σ(*I*)
Graphite monochromator	*R*_int_ = 0.054
φ and ω scans	θ_max_ = 29.1°, θ_min_ = 2.4°
Absorption correction: multi-scan (*SADABS*; Bruker, 2010)	*h* = −13→14
*T*_min_ = 0.86, *T*_max_ = 0.95	*k* = −14→14
30647 measured reflections	*l* = −22→22

### Refinement

**Table:**

Refinement on *F*^2^	Secondary atom site location: difference Fourier map
Least-squares matrix: full	Hydrogen site location: inferred from neighbouring sites
*R*[*F*^2^ > 2σ(*F*^2^)] = 0.034	H-atom parameters constrained
*wR*(*F*^2^) = 0.097	*w* = 1/[σ^2^(*F*_o_^2^) + (0.0474*P*)^2^ + 1.204*P*] where *P* = (*F*_o_^2^ + 2*F*_c_^2^)/3
*S* = 1.07	(Δ/σ)_max_ < 0.001
2475 reflections	Δρ_max_ = 0.38 e Å^−^^3^
129 parameters	Δρ_min_ = −0.39 e Å^−^^3^
0 restraints	Extinction correction: *SHELXL97* (Sheldrick, 2008), Fc^*^=kFc[1+0.001xFc^2^λ^3^/sin(2θ)]^-1/4^
Primary atom site location: structure-invariant direct methods	Extinction coefficient: 0.0061 (7)

### Special details

**Table:**

Geometry. All e.s.d.'s (except the e.s.d. in the dihedral angle between two l.s. planes) are estimated using the full covariance matrix. The cell e.s.d.'s are taken into account individually in the estimation of e.s.d.'s in distances, angles and torsion angles; correlations between e.s.d.'s in cell parameters are only used when they are defined by crystal symmetry. An approximate (isotropic) treatment of cell e.s.d.'s is used for estimating e.s.d.'s involving l.s. planes.
Refinement. Refinement of *F*^2^ against ALL reflections. The weighted *R*-factor *wR* and goodness of fit *S* are based on *F*^2^, conventional *R*-factors *R* are based on *F*, with *F* set to zero for negative *F*^2^. The threshold expression of *F*^2^ > σ(*F*^2^) is used only for calculating *R*-factors(gt) *etc*. and is not relevant to the choice of reflections for refinement. *R*-factors based on *F*^2^ are statistically about twice as large as those based on *F*, and *R*- factors based on ALL data will be even larger. H-atoms were placed in calculated positions (C—H = 0.95 - 0.98 Å) and included as riding contributions with isotropic displacement parameters 1.2 - 1.5 times those of the attached carbon atoms.

### Fractional atomic coordinates and isotropic or equivalent isotropic displacement parameters (Å^2^)

**Table:**

	*x*	*y*	*z*	*U*_iso_*/*U*_eq_	
N1	0.07438 (11)	0.87020 (10)	0.65230 (6)	0.0173 (2)	
N2	−0.23590 (12)	0.56153 (11)	0.49548 (7)	0.0259 (3)	
C1	0.15878 (14)	0.95747 (13)	0.69622 (9)	0.0238 (3)	
H1A	0.2307	0.9125	0.7215	0.036*	
H1B	0.1071	0.9994	0.7372	0.036*	
H1C	0.1944	1.0183	0.6590	0.036*	
C2	−0.04511 (13)	0.90672 (12)	0.62884 (8)	0.0201 (3)	
H2	−0.0730	0.9884	0.6394	0.024*	
C3	−0.12714 (13)	0.82710 (12)	0.58970 (8)	0.0198 (3)	
H3	−0.2115	0.8530	0.5731	0.024*	
C4	−0.08458 (13)	0.70782 (12)	0.57475 (7)	0.0175 (3)	
C5	0.03964 (13)	0.67124 (12)	0.59898 (8)	0.0204 (3)	
H5	0.0700	0.5902	0.5887	0.024*	
C6	0.11766 (14)	0.75535 (13)	0.63820 (8)	0.0200 (3)	
H6	0.2026	0.7320	0.6554	0.024*	
C7	−0.16859 (13)	0.62451 (12)	0.53131 (8)	0.0201 (3)	
Cl1	0.01260 (3)	0.73669 (3)	0.35515 (2)	0.01939 (12)	
O1	0.12396 (10)	0.79937 (10)	0.32127 (6)	0.0253 (2)	
O2	−0.05193 (12)	0.81513 (10)	0.41181 (6)	0.0285 (3)	
O3	0.05528 (12)	0.62622 (10)	0.39448 (9)	0.0386 (3)	
O4	−0.07787 (11)	0.70787 (15)	0.29216 (7)	0.0432 (4)	

### Atomic displacement parameters (Å^2^)

**Table:**

	*U*^11^	*U*^22^	*U*^33^	*U*^12^	*U*^13^	*U*^23^
N1	0.0169 (5)	0.0170 (5)	0.0180 (5)	−0.0015 (4)	0.0016 (4)	0.0016 (4)
N2	0.0280 (6)	0.0231 (6)	0.0265 (6)	−0.0034 (5)	−0.0028 (5)	0.0021 (5)
C1	0.0233 (6)	0.0201 (6)	0.0278 (7)	−0.0042 (5)	−0.0028 (5)	−0.0017 (5)
C2	0.0206 (6)	0.0157 (6)	0.0241 (6)	0.0022 (5)	0.0010 (5)	0.0026 (5)
C3	0.0177 (6)	0.0184 (6)	0.0232 (6)	0.0023 (5)	−0.0001 (5)	0.0039 (5)
C4	0.0187 (6)	0.0182 (6)	0.0155 (5)	−0.0011 (5)	0.0017 (4)	0.0020 (4)
C5	0.0200 (6)	0.0183 (6)	0.0229 (6)	0.0040 (5)	0.0013 (5)	−0.0013 (5)
C6	0.0160 (6)	0.0210 (6)	0.0229 (6)	0.0030 (5)	0.0007 (5)	0.0007 (5)
C7	0.0208 (6)	0.0184 (6)	0.0211 (6)	0.0010 (5)	0.0005 (5)	0.0041 (5)
Cl1	0.01679 (17)	0.01989 (18)	0.02150 (19)	0.00045 (11)	0.00104 (11)	−0.00297 (11)
O1	0.0221 (5)	0.0261 (5)	0.0277 (5)	−0.0036 (4)	0.0033 (4)	0.0018 (4)
O2	0.0401 (6)	0.0236 (5)	0.0217 (5)	0.0050 (4)	0.0099 (4)	−0.0012 (4)
O3	0.0292 (6)	0.0213 (5)	0.0654 (9)	0.0055 (4)	0.0110 (6)	0.0133 (5)
O4	0.0193 (5)	0.0806 (10)	0.0297 (6)	−0.0081 (6)	−0.0003 (5)	−0.0213 (6)

### Geometric parameters (Å, º)

**Table:**

N1—C2	1.3443 (18)	C3—H3	0.9500
N1—C6	1.3458 (17)	C4—C5	1.3924 (19)
N1—C1	1.4793 (17)	C4—C7	1.4456 (18)
N2—C7	1.1420 (18)	C5—C6	1.3806 (19)
C1—H1A	0.9800	C5—H5	0.9500
C1—H1B	0.9800	C6—H6	0.9500
C1—H1C	0.9800	Cl1—O2	1.4373 (10)
C2—C3	1.3728 (19)	Cl1—O3	1.4382 (12)
C2—H2	0.9500	Cl1—O4	1.4389 (12)
C3—C4	1.3907 (18)	Cl1—O1	1.4441 (10)
			
C2—N1—C6	121.46 (12)	C3—C4—C7	119.27 (12)
C2—N1—C1	119.16 (11)	C5—C4—C7	120.72 (12)
C6—N1—C1	119.36 (11)	C6—C5—C4	118.54 (12)
N1—C1—H1A	109.5	C6—C5—H5	120.7
N1—C1—H1B	109.5	C4—C5—H5	120.7
H1A—C1—H1B	109.5	N1—C6—C5	120.52 (12)
N1—C1—H1C	109.5	N1—C6—H6	119.7
H1A—C1—H1C	109.5	C5—C6—H6	119.7
H1B—C1—H1C	109.5	N2—C7—C4	177.94 (14)
N1—C2—C3	120.65 (12)	O2—Cl1—O3	109.38 (7)
N1—C2—H2	119.7	O2—Cl1—O4	108.60 (7)
C3—C2—H2	119.7	O3—Cl1—O4	110.50 (9)
C2—C3—C4	118.86 (12)	O2—Cl1—O1	110.03 (7)
C2—C3—H3	120.6	O3—Cl1—O1	109.56 (7)
C4—C3—H3	120.6	O4—Cl1—O1	108.76 (7)
C3—C4—C5	119.97 (12)		
			
C6—N1—C2—C3	0.2 (2)	C3—C4—C5—C6	0.48 (19)
C1—N1—C2—C3	−178.38 (12)	C7—C4—C5—C6	178.09 (12)
N1—C2—C3—C4	0.0 (2)	C2—N1—C6—C5	−0.1 (2)
C2—C3—C4—C5	−0.4 (2)	C1—N1—C6—C5	178.49 (12)
C2—C3—C4—C7	−178.02 (12)	C4—C5—C6—N1	−0.3 (2)

### Hydrogen-bond geometry (Å, º)

**Table:**

*D*—H···*A*	*D*—H	H···*A*	*D*···*A*	*D*—H···*A*
C1—H1*A*···O4^i^	0.98	2.37	3.245 (2)	149
C1—H1*C*···O2^ii^	0.98	2.61	3.2540 (19)	123
C2—H2···O1^ii^	0.95	2.46	3.4001 (18)	173
C2—H2···O2^ii^	0.95	2.63	3.2549 (18)	123
C3—H3···N2^iii^	0.95	2.67	3.3098 (18)	125
C3—H3···O3^iv^	0.95	2.46	3.300 (2)	148
C6—H6···O4^i^	0.95	2.50	3.351 (2)	149

Symmetry codes: (i) *x*+1/2, −*y*+3/2, −*z*+1; (ii) −*x*, −*y*+2, −*z*+1; (iii) −*x*−1/2, *y*+1/2, *z*; (iv) *x*−1/2, −*y*+3/2, −*z*+1.
